# Reusability and stability of a novel ternary (Co–Cd–Fe)-LDH/PbI_2_ photoelectrocatalytst for solar hydrogen production

**DOI:** 10.1038/s41598-021-85005-y

**Published:** 2021-03-10

**Authors:** Fatma Mohamed, Nour Bhnsawy, Mohamed Shaban

**Affiliations:** 1 Materials Science Laboratory, Chemistry Department, Faculty of Science, Beni-Suef University, Beni-Suef, 62514 Egypt; 2 Nanophotonics and Applications (NPA) Lab, Faculty of Science, Beni-Suef University, Beni-Suef, 62514 Egypt; 3 Department of Physics, Faculty of Science, Islamic University in Almadinah Almonawara, Almadinah Almonawara, 42351 Saudi Arabia

**Keywords:** Environmental sciences, Chemistry, Energy science and technology, Engineering

## Abstract

The design of highly active and cost*-*effective photoelectrocatalysts for effective hydrogen generation becomes a mandatory issue due to the demands on sustainable solar fuels. Herein a novel ternary Co–Cd–Fe LDH/PbI_2_ nanocomposite (T-LDH/PbI_2_NC) was fabricated by combining strategies of doping and in-situ loading of ternary Co–Cd–Fe LDH. The morphological, structural, and optical properties of PbI_2_, T-LDH, and T-LDH/PbI_2_ NC were studied by different techniques. LDH narrows the bandgap of the nanocomposite to 2.53 eV which prolongs the lifetime of the photo-induced electrons. Subsequently, the use of T-LDH/PbI_2_ NC improves the photoelectrocatalytic (PEC) H_2_ production rate. T-LDH/PbI_2_ NC shows a catalytic H_2_ production rate of 107.53 mmol h^−1^ cm^−2^ with IPCE% of 83.8% for 307 nm and 67.3% for 508 nm. The ABPE% reaches its supreme of 4.24% for − 0.58 V and 5.41% for − 0.97 V, these values are the highest values yet for LDH-based photocatalysts. The influences of the operating temperature and monochromatic illumination on the PEC performance were studied. Also, the electrochemical surface area, thermodynamic parameters, and Tafe slopes are calculated to label the hydrogen evolution mechanism. Moreover, the stability and reusability of the T-LDH/PbI_2_ NC photoelectrode were investigated. This work not only illustrated a simplistic and accessible way to produce a new category of highly efficient photocatalysts compared to the previously reported LDH-based PEC catalysts but also demonstrates a new point of view for improving PEC performance towards industrial water splitting under sunlight irradiation.

## Introduction

Increasing demands for energy and elevating the environmental crisis have inspired researchers to develop low-cost, environmentally friendly, and reasonable sources of energy. Water splitting through the photoelectrochemical (PEC) route is considered as one of the promising approaches to produce hydrogen as chemical fuel^1,2^. The PEC water splitting process needs semiconductor photocatalysts to convert sunlight photons directly to hydrogen molecules as clean fuels. The semiconducting materials require a remarkable performance in sunlight absorption, electrons-holes separation, and electrons/holes mobility. But still, the electron–hole recombination is the main challenge in the choice of photocatalyst for PEC^3,4^. Layered-metal halides (for example; PbI_2_ and CdI_2_) are concerned with increasing interest in electrocatalysis because of their uses in the design of perovskite halides. These perovskite structures offered noticeable photoelectrocatalytic performances^5^ PbI_2_ offers high photoelectrocatalytic performances among the different SC materials however it necessitates illumination with photons of wavelengths less than ∼ 350 nm (absorption band onset). It has a bandgap wider than 3.10 eV. Then, its performance under visible light is limited. As a result, the modification of PbI_2_ using co-catalysts with suitable bandgaps for the visible light photons is considered the most common method to improve its photocatalytic hydrogen evolution (PHE) efficiency^6,7^.

Layered double hydroxides (LDH) have attracted much attention because of their high layer charge density along with two-dimensional interlayer spaces of height, which are available for generating a rational path for charge conveying^8^. NiFe, ZnCr, CoAl-LDH, and NiAl-LDH were used as co-catalysts in the field of water splitting to generate O_2_ and H_2_^9–14^. Zhang et al. has designed a modular catalyst of Ni–MgO–Al_2_O_3_ via the template of NiMgAl-LDH. He showed excellent coke- and wintering-resistance in the drying of methane reaction^15^. Kulamani et al.^16^ has reported that NiFe-LDH/g-C_3_N_4_ photocatalyst shows excellent photoelectrocatalytic performances for water splitting reactions. These remarkable photocatalytic performances on materials based on LDH render them a rational platform for exploring novel and efficient photocatalysts^17–21^.

In this work, we report for the first time a novel photoelectrocatalyst containing (Co–Cd–Fe) LDH and PbI_2_ to form T-LDH/PbI_2_ nanocomposite. Different characterization instruments have been used to investigate the properties (structures, morphologies, compositions, and optical and photoelectrocatalytic properties) of the prepared photoelectrodes. Co–Cd–Fe LDH, PbI_2,_ and T-LDH/PbI_2_ NC photoelectrodes are deposited on the surface of a graphite substrate and used for H_2_ generation through PEC water splitting. The PEC performance was evaluated in terms of electrode stability, reusability, optical filter effect, temperature effect, conversion efficiencies, Tafel slopes, and electrochemical surface area (ECSA). Finally, the number of evaluated hydrogen moles are calculated.

## Results and discussion

### Samples characterizations

#### Structural properties

**Figure 1:**
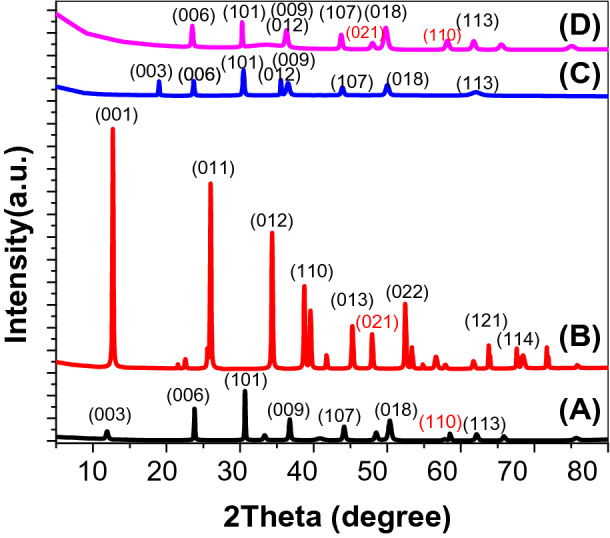
XRD of Co–Cd–Fe LDH (**A**), PbI_2_ (**B**), and T-LDH/PbI_2_ (**C**). T-LDH/PbI_2_ NC after ten runs in PEC (**D**).

XRD charts of (Co–Cd–Fe) LDH, PbI_2_ and T-LDH/PbI_2_ NC are displayed in Fig. 1A–D. The XRD chart of the (Co–Cd–Fe)LDH appears highly similar to the hexagonal phase of the hydrotalcite LDH (Fig. 1A). The observed XRD peaks referred to as the diffractions from (003), (006), (101), (009), (107), (018), (110), and (113) planes of a usual LDH^22^. The high reflection intensity of these peaks shows the high crystallinity of the studied LDH. However, the diffraction peaks of the lead iodide appear at 2 theta = 12.6°, 25.9°, 34.2°, 39.5°, 45.2°, 47.5°, 53.2°, 63.7° and 67.5° (Fig. 1B). These peaks can be respectively indexed to (001), (011), (012), (110), (013), (021), (022), (121), and (114) of PbI_2_ (ICDD Card No. 04-007-3845). The observed chart of PbI_2_ agreed well with previously observed charts in literature^23^.

The mean crystallites sizes (D_C_) of PbI_2_ were estimated utilizing Scherrer's relation. The calculated mean crystallites size was ~ 70 nm^24^. The mean value of the microstrain for PbI_2_ was ~ 0.2%. The dislocations density (δ_d_ = N/D_C_^2^, N is constant) was also estimated to evaluate the density of defects and the quality of the crystal. The smallest δ_d_ for PbI_2_ was calculated when N = 1^25^. The obtained value of δ_d_ is 2.05 × 10^−4^ that refers to the high quality of the synthesized PbI_2_ crystal_._

The XRD chart of T-LDH/PbI_2_ illustrates the main XRD peaks of the (Co–Cd–Fe)LDH but with observed shifts in the position (Fig. 1C). Also, a mixed-phase between PbI_2_ and LDH appears in this pattern. The observed XRD peaks are referred to diffractions from (003), (006), (101), (012), (009), (107), (018), and (113) planes. Moreover, the significant rise in the XRD peaks intensities refers to the distribution of PbI_2_ on LDH layers and the good distribution of PbI_2_ between the layers of LDH.

#### Morphologies of the samples

**Figure 2:**
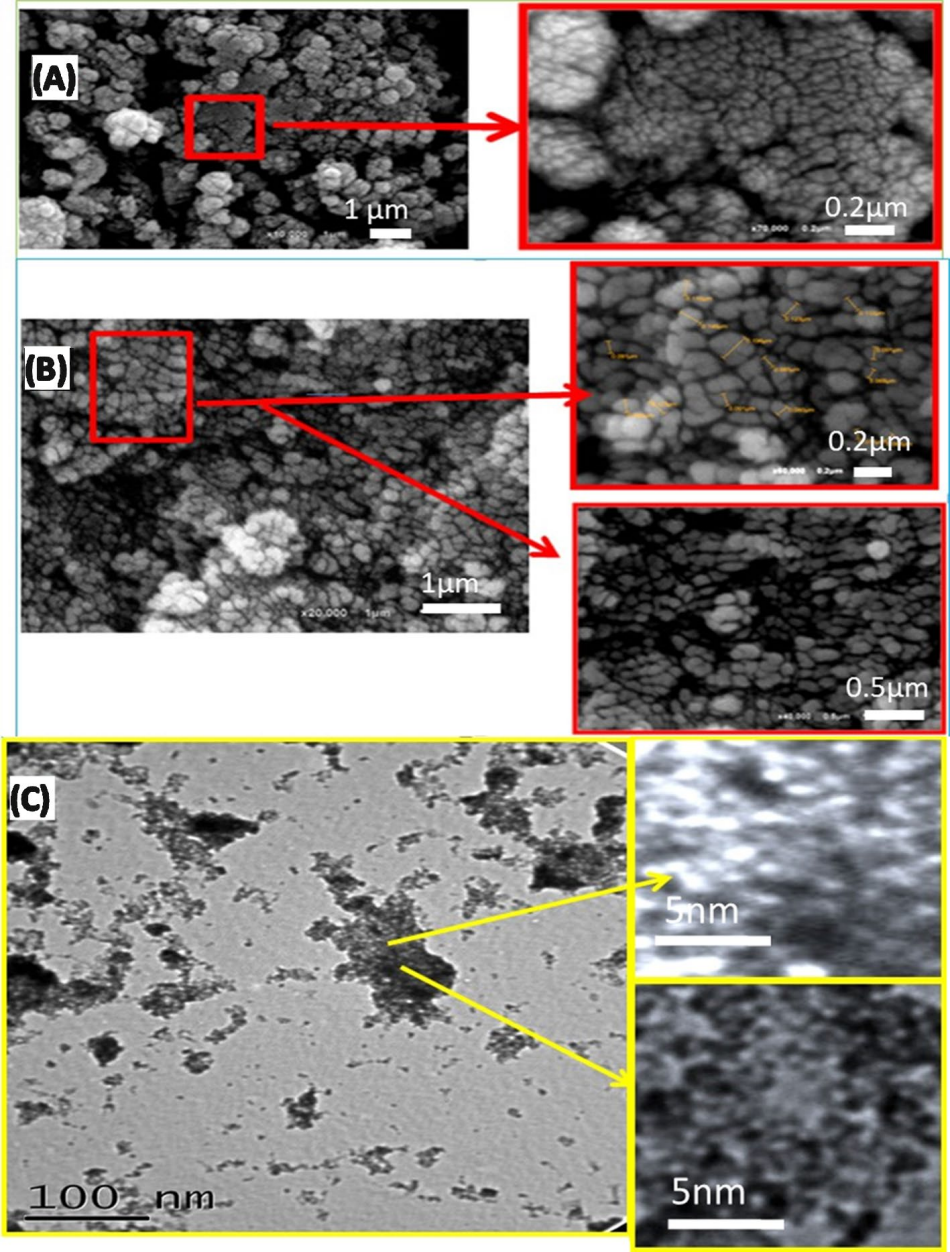
FE-SEM of Co–Cd–Fe LDH (**A**) and T-LDH/PbI_2_ NC (**B**); and TEM images of nanocomposite (**C**).

The morphologies of (Co–Cd–Fe)LDH and T-LDH/PbI_2_ NC were investigated using FE-SEM and TEM (Fig. 2). The prepared (Co–Cd–Fe)LDH looks like agglomerated nanoparticles stacked together, Fig. 2A. These particles have non-uniform shapes with different sizes. These particles are subsequently folded as our brains. A close examination of the sample using a high magnification SEM image reveals the existence of many small nano protrusions on the surface of LDH particles. After the formation of PbI_2_ on Co–Cd–Fe LDH, PbI_2_ distributes between the layers of LDH particles and increases the porosity of LDH. As a result, the surface area has increased and the particle size distribution was found to be 70 ± 10 nm, Fig. 2B. A TEM micrograph of T-LDH/PbI_2_ NC, Fig. 2C, illustrates the presence of PbI_2_ particles on Co–Cd–Fe LDH particles. It is seen that the layered structure of the LDH prevents the agglomeration of the PbI_2_ particles. This is useful for the separation of photogenerated electrons and holes. Hence, it is highly expected that this photocatalyst can be applied efficiently for photoelectrochemical hydrogen generation. The nanoporous features of the nanocomposite are shown in the magnified images of Fig. 2C.

**Figure 3:**
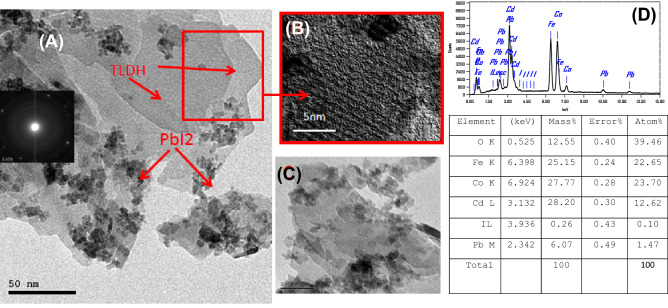
HR-TEM of T-LDH/PbI_2_NC at different magnifications (**A**–**C**) and EDX of T-LDH/PbI_2_ (**D**).

Figure 3A–C shows high resolution-transmission electron microscopy (HR-TEM) images of T-LDH with PbI_2_. The distributions of PbI_2_ particles on the Co–Cd–Fe-LDH platelets are clearly observed. The highly magnified HR-TEM images were shown in Fig. 3B,C are used to confirm the fine structure of T-LDH/PbI_2_ NC, which showed the stacking of the layered nanosheets. The Co–Cd–Fe LDH component showed a plate-like morphology. The selected area electron diffraction (SAED) pattern illustrates the existence of the diffraction rings, inset of Fig. 3A, these rings confirmed the polycrystalline state and homogeneous distribution of PbI_2_ on the Co–Cd–Fe LDH layers. These results may enhance the ECSA-value and improve the separation of interfacial charge transfer between Co–Cd–Fe-LDH and PbI_2_ particles.

The EDX spectrum, Fig. 3D, of T-LDH/PbI_2,_ and the inserted quantitative analysis Table in Fig. 3 clearly indicate the presence of cobalt, iron, and cadmium signal within the walls. The molar ratios of Co:Cd:Fe were found to be approximately 1:1:1. These ratios are in good agreement with the ratios used during the preparation of T-LDH.

#### Function groups identification

**Figure 4:**
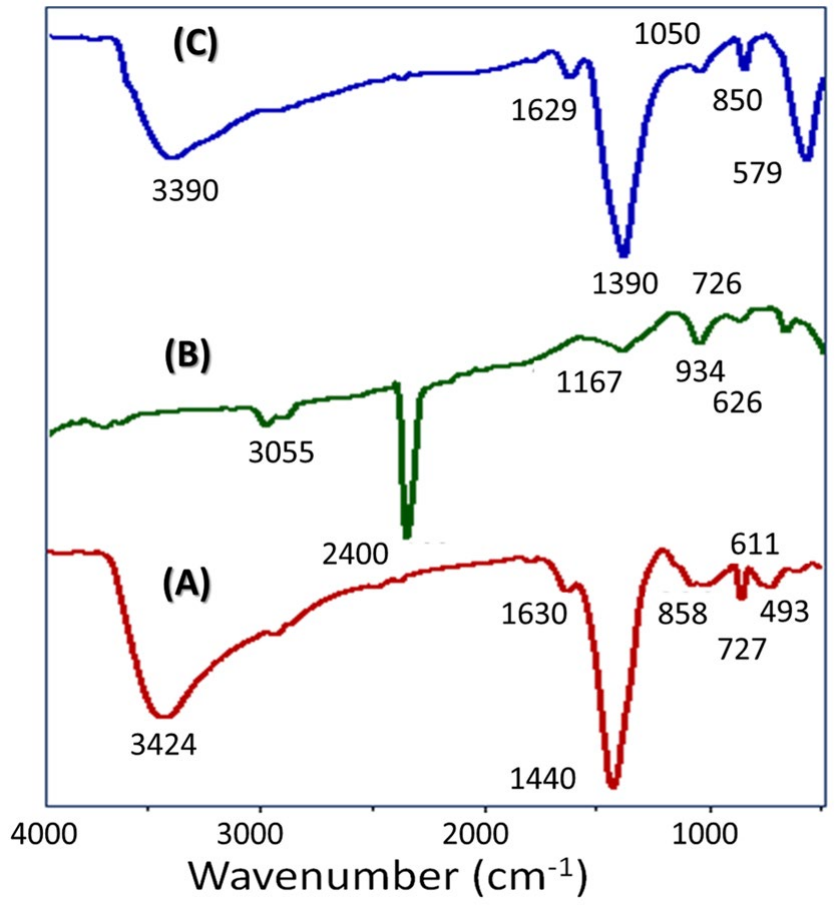
FTIR spectra of Co–Cd–Fe LDH (**A**), PbI_2_ (**B**), and T-LDH/PbI_2_ NC (**C**).

The FTIR charts of (Co–Cd–Fe)LDH, PbI_2,_ and T-LDH/PbI_2_ NC are displayed in Fig. 4A–C. For (Co–Cd–Fe)LDH, the identified band at 3424 cm^−1^ is familiar to the OH-stretching of LDH and the H_2_O interlayer^25^. The observed peak close to 1630 cm^−1^ refers to the O–H bending and 1350 cm^−1^ refers to the bending mode of the H_2_O molecule. While the peak that appears at 1430 cm^−1^ is ascribed to the NO^3−^-stretching mode^26^. The noticed modes below 1000 cm^−1^ are assigned to the M–O vibrations of LDH Fig. 4A. The FTIR spectral modes of PbI_2_, Fig. 4B, are ascribed to vibrations of inorganic clusters. The mode at 1490 cm^−1^ is credited to the H_2_O molecule's absorption^27^. Figure 4B confirms the presence of strong interactions between Pb–I clusters. Symmetric and asymmetric modes are observed at 3055 and 3700 cm^−1^ of the Pb–I^28^. The peak at 2400 cm^−1^ was ascribed to the water stretching region. After the combination, the FTIR spectrum of T-LDH/PbI_2_ NC exhibits a shift to lower wavenumbers (redshift) which may be ascribed to the distribution of Pb–I_2_ into layers of LDH was shown at Fig. 4C.

#### Optical properties of Co–Cd–Fe LDH, PbI_2_, and T-LDH/PbI_2_ NC

**Figure 5:**
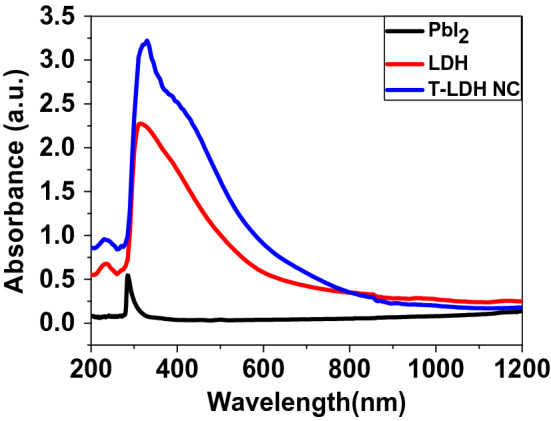
Absorbance spectra of PbI_2_, Co–Cd–Fe LDH, and T-LDH/PbI_2_ NC.

The light absorption property of all samples is explored by UV–Vis absorption spectra as displayed in Fig. 5. The absorption edge of pure PbI_2_ appears at 300 nm. No important features are observed in the Vis/IR regions. This was due to its intrinsic bandgap absorption Fig. 5. After the growth of T-LDH/PbI_2_ NC, the absorption edge of Co–Cd–Fe LDH redshifts to the visible-region. So, a noticeable improvement in absorption can be observed in Fig. 5. Also, the layers of LDH facilitate the motion of the photo-produced electrons^29^. As well, the T-LDH/PbI_2_ NC showed a wider absorption band in the visible-light-region. These enhanced absorption capabilities result from the extension of the band to cover a broad region of the incident photons (300–800 nm). This range represents > 43% of sunlight at the Earth's surface. Therefore, T-LDH/PbI_2_ NC can efficiently be used as a key material for different solar energy applications including the PEC hydrogen generation. The light absorption by T-LDH/PbI_2_ NC around 500 nm is assigned to the metal–metal charges transfer that contributes efficiently to the PEC H_2_O splitting Fig. 5 i.e., the enhanced absorption capabilities toward the visible photons is accredited to electron transfer from the PbI_2_ conduction band to the LDH surface. Therefore, the absorption edge of T-LDH/PbI_2_ NC is observed in the visible-light-region, which leads to the reduction of the bandgap energy, Fig. 5.

**Figure 6:**
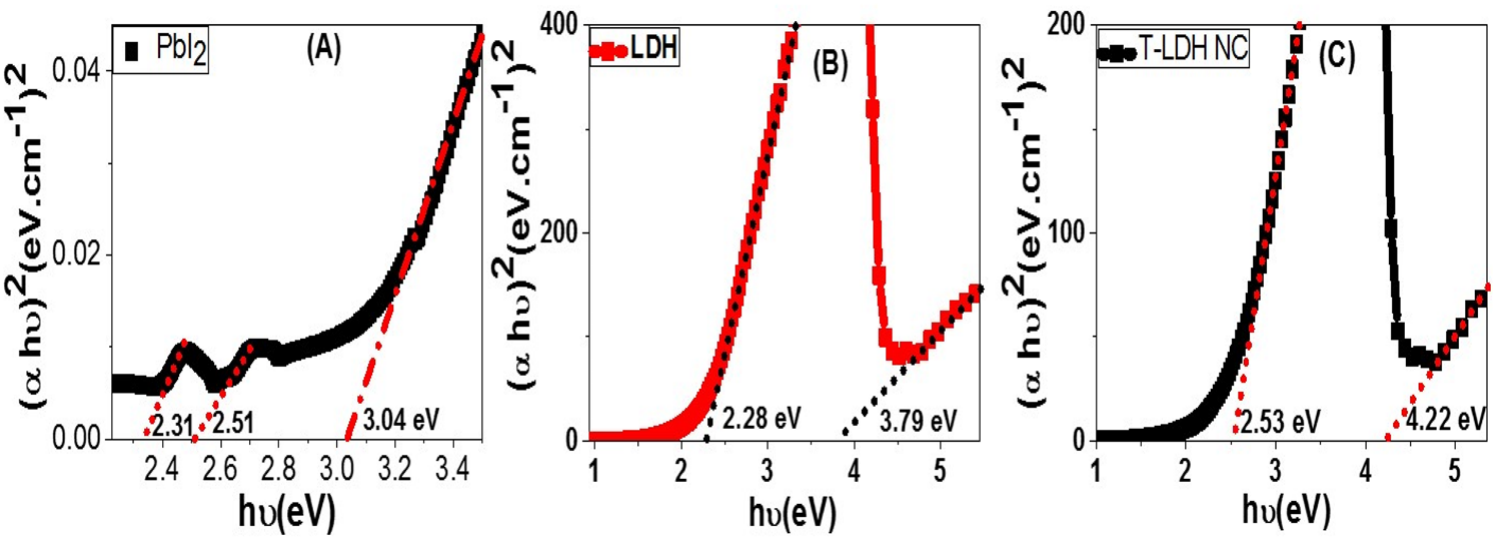
(αhʋ)^2^ vs. hʋ for energy gap calculation of **(A)** PbI_2_, **(B)** Co–Cd–Fe LDH, and **(C)** T-LDH/PbI_2_ NC.

Based on the UV–visible absorbance spectra (Fig. 6), the optical band gap of PbI_2_, Co–Cd–Fe LDH, and T-LDH/PbI_2_ NC can be obtained utilizing the absorption values (A) and absorption coefficient (*α*_*A*_) according to Eq. (1) (Tauc relation)^1^;

1 $$\alpha_{A} E_{ph} = {\text{A}}\left( {E_{ph} - E_{g} } \right)^{{{1}/{2}}}$$

wherever *E*_*ph *= _*hν* and *E*_*g*_ are for the photon energy and bandgap energy. The values of *α*_*A*_ are obtained from Eq. (2)^30,31^:

2 $$\alpha_{A} = { 2}.{3}0{3 } \times { 1}0^{{3}} A \, \beta /\ell {\text{ C}}_{{\text{p}}}$$

wherever $\beta$, $\ell$, and C_p_ are the material density, quartz cell width (1 cm), and suspended material concentration.

The bandgap energies were determined by inferring the linear portion of (α_*A*_* E*_*ph*_)^2^–*E*_*ph*_ plot with the *E*_*ph*_-axis, Fig. 6.

The bandgap energy is estimated to be 3.04 eV for PbI_2_, which agreed to the previously stated bandgap values for nanostructure PbI_2_ (> 2 eV). This bandgap is originated from Pb *s* to Pb *p* interband transitions was shown in Fig. 6A. Also, there are two other bands at 2.31 and 2.51 eV due to the existence of two discrete absorption plasmons as a result of the quantum confinement effects and the orbital hybridizations between the I_*p*-orbitals_ and Pb_*s*-orbitals_. On the other hand, the bandgap values are estimated from to be 2.28 and 2.53 eV for Co–Cd–Fe LDH, and T-LDH/PbI_2_ NC; respectively Fig. 6B,C. Figures 5 and 6 refer to the improvement of the optical absorption and the redshift of the optical band gap of LDH due to the incorporation of PbI_2_ to form T-LDH/PbI_2_ NC. So it is highly expected that T-LDH/PbI_2_ NC can be more effective than its constituents for applying in PEC H_2_ generation. In Fig. 6B, the Co–Cd–Fe LDH showed the existence of two band gaps at 2.28 and 3.79 eV. Whereas the T-LDH/PbI_2_ NC band gaps are shifted to 2.53 and 4.22 eV, Fig. 6C. The blue shift of the band gap and the absorption edge is ascribed to the Burstein–Moss shift; whereas the electronic transitions from oxygen *2p* to metal *ns* or *np* levels enhance the possibility of filling the bottom of the conduction band with electrons. Based on the exclusion principle this leads to blue shifts in the optical absorption band position. Based on that, the bandgap broadening and the intensive absorbance of the visible-light photons of T-LDH/PbI_2_ NC as compared to the pure LDH will facilitate the electronic transitions and offer more electron/hole pairs under sunlight illumination. All of these encourage us to apply the designed T-LDH/PbI_2_ NC for photoelectrochemical H_2_O-splitting under sunlight^32^.

### PEC properties of T-LDH/PbI_2_ NC

#### Effect of photocatalyst and white light illumination

**Figure 7:**
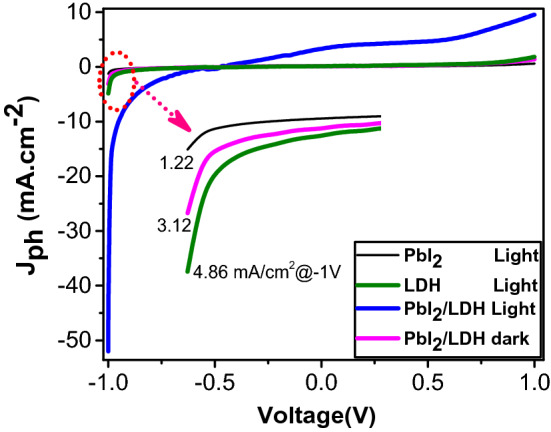
photocurrent density–voltage (J_ph_–V) curves of PEC reaction under dark and white light illumination using 0.3 M KOH utilizing PbI_2_, Co–Cd–Fe LDH, and T-LDH/PbI_2_ NC.

The efficiency of PEC depends on the photo responsive of the catalyst. So we measured the PEC characteristics for the three electrodes; PbI_2_, Co–Cd–Fe LDH, and T-LDH/PbI_2_ NC for determining the different current densities (J_ph_) for photo electrodes. The photocurrent density–voltage (J_ph_–V) curves are performed with increment 1 mVs^−1^ in 0.3 M KOH (100 ml) solution at 25 °C was shown at Fig. 7. The surface area of two electrodes (working and counter) electrodes are 1 cm^2^. A light power is adjusted to be 100 mW cm^−2^ with aid of 500 W Meitrcury-Xenon light source (Newport, MODEL: 66926-500HX-R07). The measured current densities for PbI_2_, Co–Cd–Fe -LDH and T-LDH/PbI_2_ NC photoelectrodes were 1.22, 4.86, and 53.27 mA cm^−2^ in − 1 V, respectively. The current density using the T-LDH/PbI_2_ NC photoelectrode generated the greater no of electrons than others under white light exposure. This is ascribed to its highly electrical surface charges and the suitable optical bandgap which in turn to increase in the absorbance in the Vis/IR range.

In pristine PbI_2_, there are decline in kinetics of water oxidation on the surface thus in valence band, the accumulation of positive holes will occur and stimulate electron/hole recombination in the conduction band, which displays low photo response^33^.

While Co–Cd–Fe LDH (TLDH) represented as an effective co-catalyst. It distinguishes with highly conductivity and faster carrier transfer which enhances the kinetic of water oxidation so helps in initiation of the removal of the photogenerated holes accumulated at the surface of system^34^.

While after introduction of Co–Cd–Fe LDH with PbI_2_, the performance of ternary T-LDH/PbI_2_ towards PEC is higher than others. Its outstanding efficiency is attributed to the synergistic effect of TLDH and PbI_2_ in the ternary T-LDH/PbI_2_.

Generally, the white light illumination has a crucial effect on PEC technique.the light stimulates the electrons of photocatalyst for hydrogen generation than dark conditions. The photocurrent was J_ph_ = 53.27 and 3.12 eV mA cm^−2^ for dark condition as shown in Fig. 6. This amazing increase is assigned to the photoexcitation of electrode and generation of charged carrier (e^−^/hole^+^ pair) which helps in water splitting and hydrogen generation^35^.

#### Tafel slopes and ECSAs of the photoelectrodes

**Figure 8:**
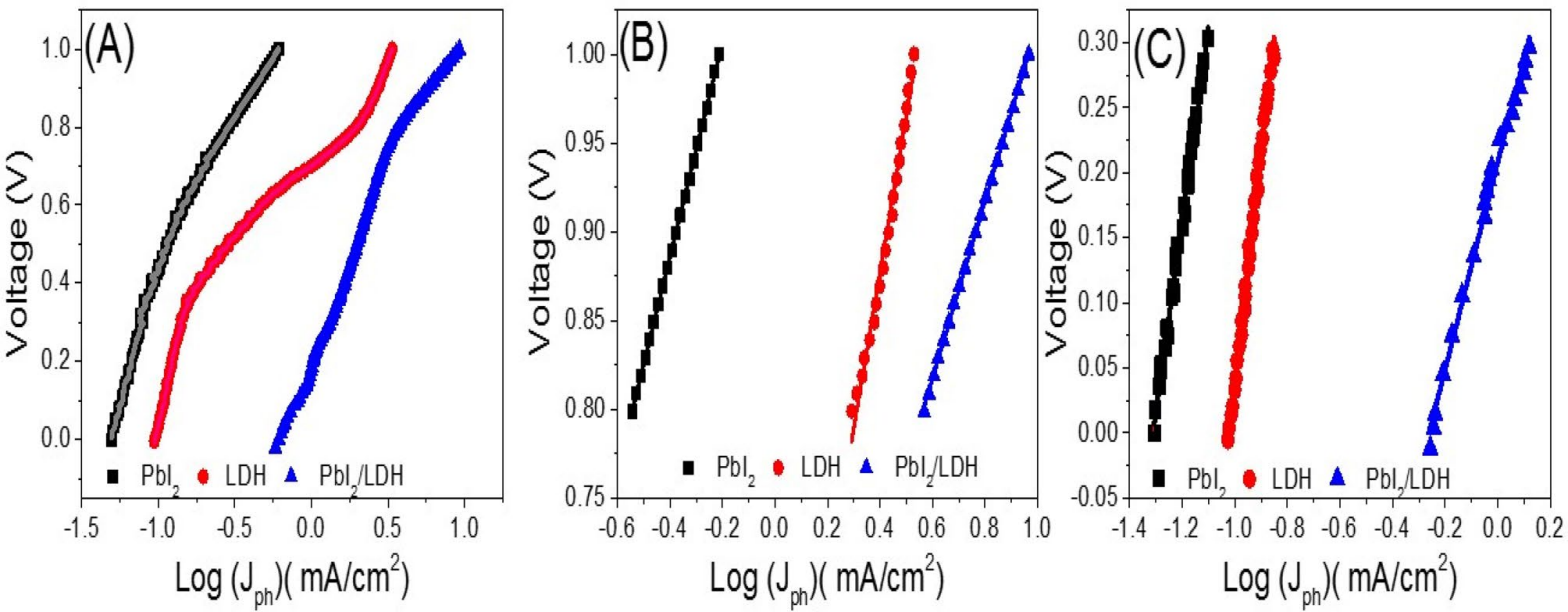
Tafel behaviors, V–log(J_ph_) (**A**–**C**), obtained in 0.3 M KOH using PbI_2_, Co–Cd–Fe LDH, and T-LDH/PbI_2_ NC.

Tafel curves, Voltage–log(J_ph_), are presented in Fig. 8A–C from the J_ph_–Voltage characteristics of Fig. 8A to mark the hydrogen evaluation reaction (HER) mechanism^36^. The Tafel slopes of the straight lines in Fig. 8B,C are represented by β1 and β2 at low and high HER potential^36^. β_1_ and β_2_ values are reported in Table 1 with their standard deviations and the correlation R^2^-coefficients. Tafel slopes of 30, 40 and 120 mV dec^−1^ apply to Volmer–Tafel (recombination is rate-limiting) mechanism, Volmer–Heyrovsky (PEC desorption is rate-limiting) mechanism, and the dependency on different reaction paths of surface coverage by adsorbed H_2_. β_1_ and β_2_ remind us of the over-potentials required to increase the HER rate by 10 folds^36^. Thus, the calculated β_1_ and β_2_, Table 1, of the T-LDH/PbI_2_ NC electrode (49.91 and 79.61 mV dec^−1^) evidenced its improved PEC characteristic in HER.

**Table 1:** β_1_ and β_2_ of the three photoelectrodes with the statistical parameters (standard deviations and correlation R^2^-coefficients).

Sample	β1 (mV dec^−1^)	R2	β2 (mV dec^−1^)	R2
PbI2	60.45 ± 0.18	0.9998	147.50 ± 2.19	0.9941
LDH	87.71 ± 2.46	0.9926	177.23 ± 2.26	0.9953
PbI2/LDH	49.91 ± 0.20	0.9997	79.61 ± 1.64	0.9924

The values of ECSAs for the three electrodes are obtained using the Randles–Sevcik equation, ECSA = I(R T/*v* D)^1/2^/[0.446 (C n F)^3/2^], where n = 1 refers to one electron contribution in the redox reaction, F and R denote to the Faraday and gas-molar constants^37^. Also, C signifies the analytes concentration, T signifies the reaction temperature, and D represents the analyte-diffusion constant^37^. Using J–V curves, Fig. 8, the ECSAs values for the three electrodes are calculated using ECSA = Q·(m·C)^−1^. Whereas Q, m, and C refer to the hydrogen-adsorption charges in the negative-scan after double-layered charges correction, photocatalyst mass, and the complete monolayer charges of hydrogen atoms, respectively, covering the electrode^38,39^. At a scanning-rate of 10 mV, the Q values are estimated using the J–V curves integrations/10 mV. The values of ECSAs of the three electrodes are 4.39, 18.98, 60.22 m^2^ g^−1^, respectively. The high value of ECSA for the T-LDH/PbI_2_ NC electrode compared to the LDH electrode and PbI_2_ electrode explains its high photocatalytic performance.

#### Reusability and stability of photoelectrode

**Figure 9:**
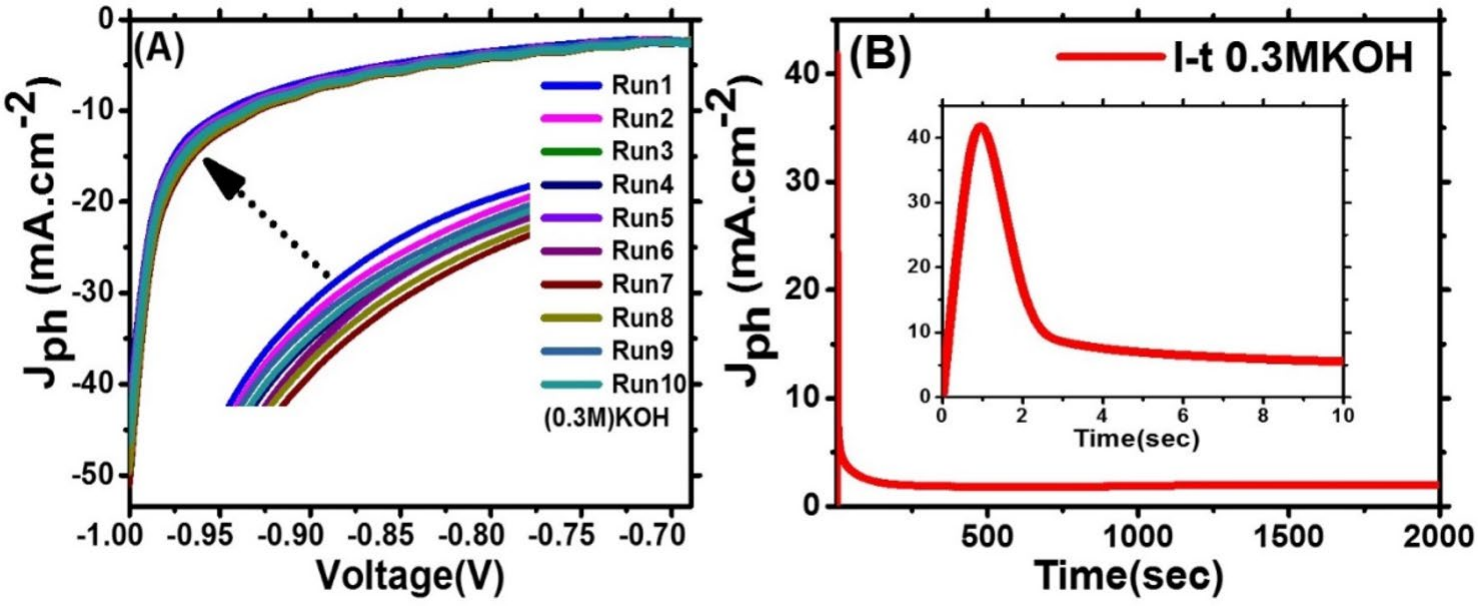
(**A**) J_ph_–voltage characteristics for different numbers of runs and (**B**) J_ph_–time characteristic in 0.9 V of PEC HER reaction under white light illumination using 0.3 M KOH and T-LDH/PbI_2_ electrode.

The photostability of T-LDH/PbI_2_ NC for PEC behaviors was studied for many runs in 0.3 M KOH as a sacrificial agent, Fig. 9A. our findings were showed that The performance of this photoelectrode is stable with a long time. From this figure, the J_ph_ of the T-LDH/PbI_2_ approximately not changed. The stability of the T-LDH/PbI_2_ photoelectrode during the hydrogen evolution reaction was investigated in 0.3 M KOH through studying the variation of the J_ph_ vs. the exposure time at constant − 0.9 V, Fig. 9B displayed the behavior of J_ph_ in a very short period (10 s) to achieve nearly a stable value of 5.5 mA cm^−2^. The dramatic decrease in this time is caused by unnoticeable corrosion that takes place in the beginning of electrolyte reaction^40,41^.

For using the faraday laws of electrolysis, The amount of produced H_2_-during a time (t) may be estimated (Eq. 3)^42^:

3 $${\mathrm{H}}_{2} (\mathrm{moles})=\underset{0}{\overset{\mathrm{t}}{\int }} {\mathrm{J}}_{\mathrm{ph}}\frac{\mathrm{dt}}{\mathrm{F}}$$

Based on the J_ph_–t characteristic, Fig. 9B, the produced number of H_2_-moles per unit area was 107.53 mmol h^−1^ cm^−2^ for T-LDH/PbI_2_ NC.

**Figure 10:**
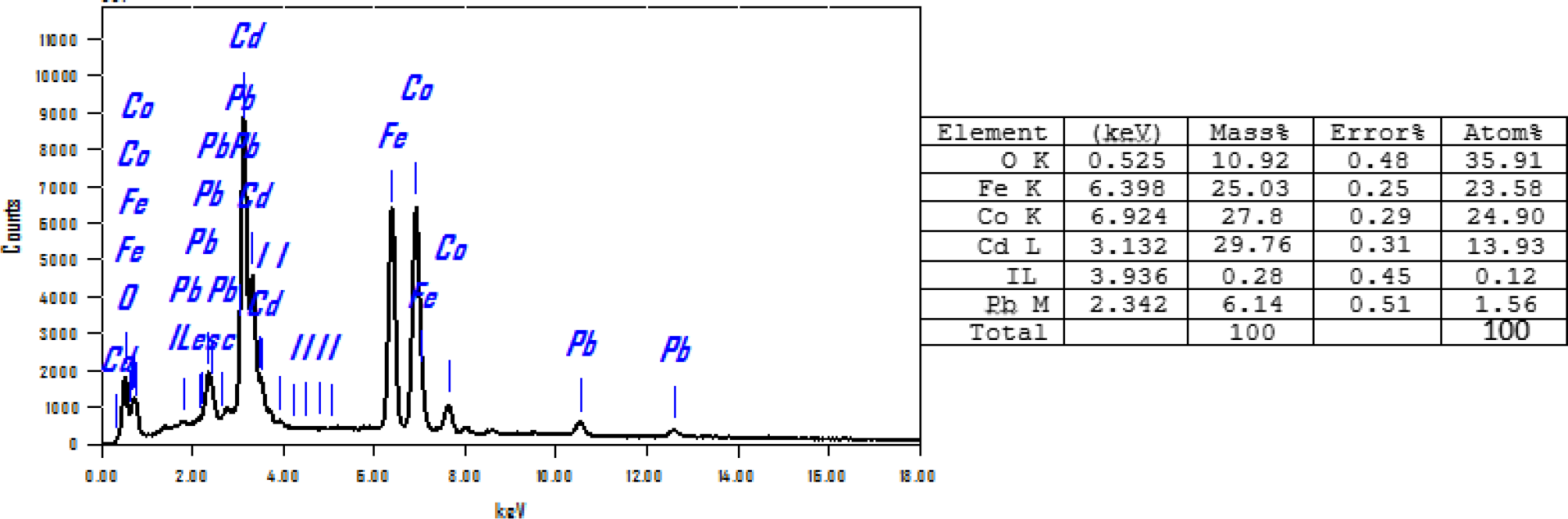
EDX of TLDH/PbI_2_NC after ten runs in KOH electrolyte for H_2_ generation.

The stability of TLDH/PbI_2_NC was further confirmed using the EDX analysis after ten runs at Fig. 10. The chemical composition of T-LDH/PbI_2_ did not change which confirms the stability of photocatalyst after many runs for H_2_ generation. But the oxygen content in the catalyst matrix was decreased from 12.55 to 10.92, which may be referred to as the generation of oxygen vacancies due to the applied potential in the presence of KOH electrolyte. Oxygen vacancies (OVs) are considered one of the defects formed in the semiconductors. These defects are generated by the removal of an oxygen atom from the catalyst matrix while it is still charged with extra electrons. OVs which diffuse at the interfaces layer in the LDH and form an interlayer of a different crystal phase due to their influence on the phase stability^40^.

The XRD analysis after ten runs can be considered as another proof of the stability of T-LDH/PbI_2_NC was shown at Fig. 1D. The structural properties of T-LDH/PbI_2_ are almost the same after many runs. The phase of the catalyst did not change which was attributed to the stability of photocatalyst after many runs for H_2_ generation. But the diffraction peak (003) disappears after ten runs for H_2_ generation which confirms the formation of oxygen vacancies. In these studies, the enhanced performance of PEC was systematically correlated with a higher density of OVs, which cause a higher Incident Photon to Current Efficiency (IPCE)^40^^.^

#### Effect of optical filters and calculation of conversion efficiencies

**Figure 11:**
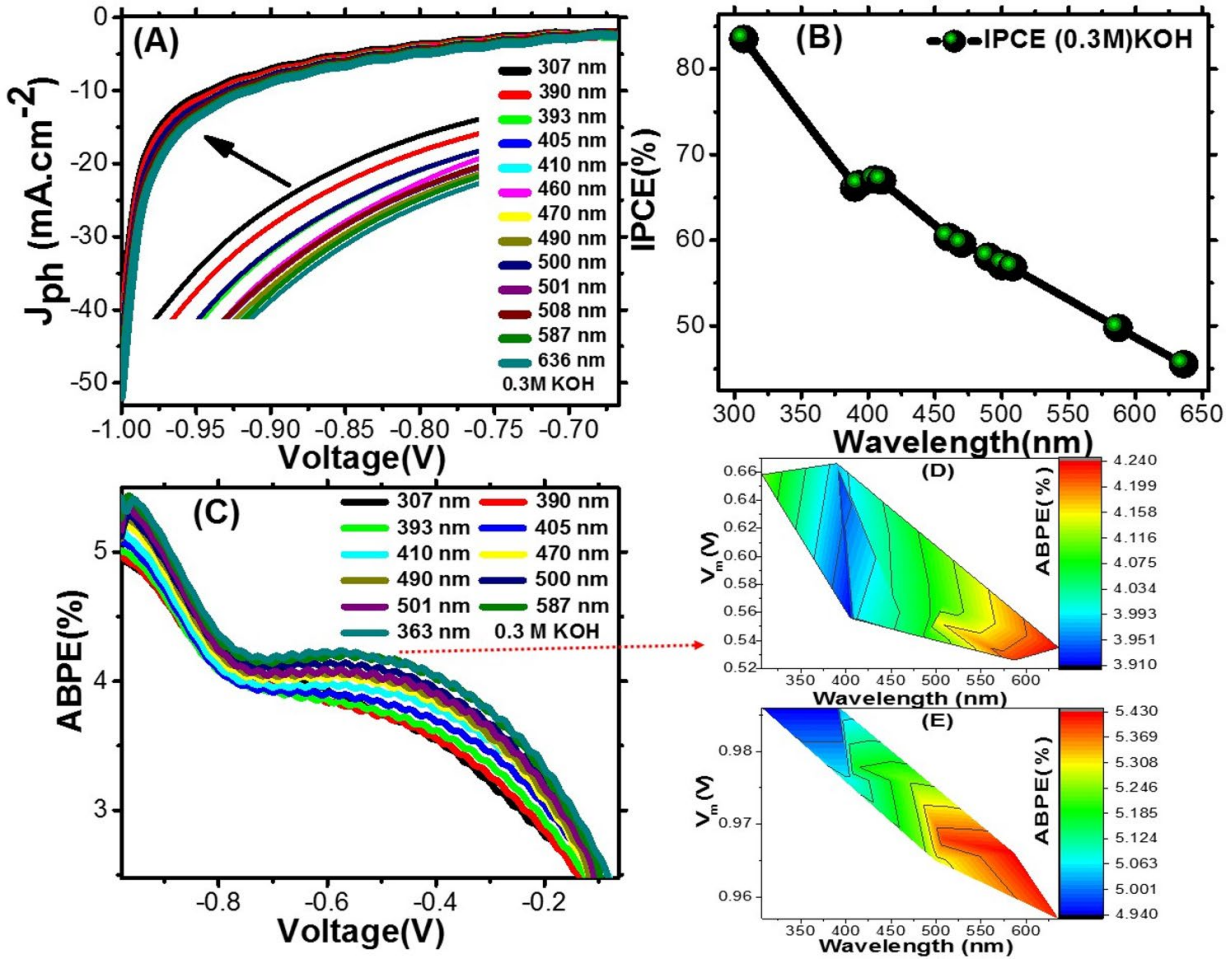
Effect of optical filters wavelength on J_ph_-voltage characteristics (**A**); IPCE% vs. wavelength in − 1 V (**B**), ABPE% vs. the applied voltage at different wavelengths (**C**), and color fill contours of the two ABPE% maximum values in the optimized voltage at different wavelengths utilizing T-LDH/PbI_2_ NC electrode in 0.3 M KOH (**D**, **E**).

Different wave length filters (307–636 nm) were applied for determination of the most suitable wave length for photoelectrode in PEC system, was represented at Fig. 11A. The PEC behaviors in 0.3 M KOH (100 ml) solution at 25 °C and increment 1 mV/at different optical filters were studied. We noticed The alteration of the monochromatic light changes the J_ph_ value. The behavior of the T-LDH/PbI_2_NC photoelectrode under the monochromatic illumination can be significant related to its absorbance response for different wave lengths light and its ability to absorb a large part of visible sunlight.

Generally, IPCE (incident-photon-to-current conversion efficacy) and ABPE (applied bias-photon-to-current efficacy) are main factors for qualifying the PEC solar hydrogen generation. IPCE value was measured at different wave length filters for determining the actual number of charge-carriers that related to the generated photocurrent per incident photon. Using the power density (P (mW cm^−2^)) and the wavelength (λ (nm)) of the monochromatic light as shown Fig. 11B, the IPCE is given using Eq. (4)^43^

4 $$\mathrm{IPCE}\left(\mathrm{\%}\right)=1240\cdot \frac{{\mathrm{J}}_{\mathrm{ph}}}{\uplambda .\mathrm{P}}\cdot 100\mathrm{\%}$$

IPCE values in − 1 V in different wave lengths is presented in Fig. 11B. The maximum IPCE for T-LDH/PbI_2_ NC photoelectrode was ~ 83% for 307 nm. Another maximum of ~ 67% was observed at 508 nm. The positional wavelengths of the two maxima are matched well with the absorption edges observed in the optical analysis of T-LDH/PbI_2_ NC, Figs. 5 and 6.

However, ABPE value describes the photo-response efficiency of a T-LDH/PbI_2_ NC electrode under an applied voltage^44,45^. ABPE can be calculated using the following equation:

5 $${\text{ABPE}}\left( \% \right) = {\text{Jph}}\frac{{\left( {1.23 - {\text{Vapp}}} \right)}}{{\uprho }} \cdot {1}00\%$$

For T-LDH/PbI_2_ NC photoelectrode in 0.3 M KOH, the ABPE% is estimated vs. the V_app_ at various wavelengths and presented in Fig. 11C. As shown in this figure, there are two maximum values of ABPE; 4.24% centered at − 0.58 V and 5.41% centered at − 0.97 V. This result may be due to the appearance of two band gaps and can be described based on the well-known photoelectric effect. This photoelectrode has a higher efficiency than the previously reported photoelectrodes^46^. The full 3D data of ABPE vs. the applied potentials and the incident wavelengths for the two maxima are presented in the color fill contours, Fig. 11D,E. The noticeable electrode response at lower potential can be advantageous for PEC cells.

#### Electrochemical impedance spectroscopy (EIS)

**Figure 12:**
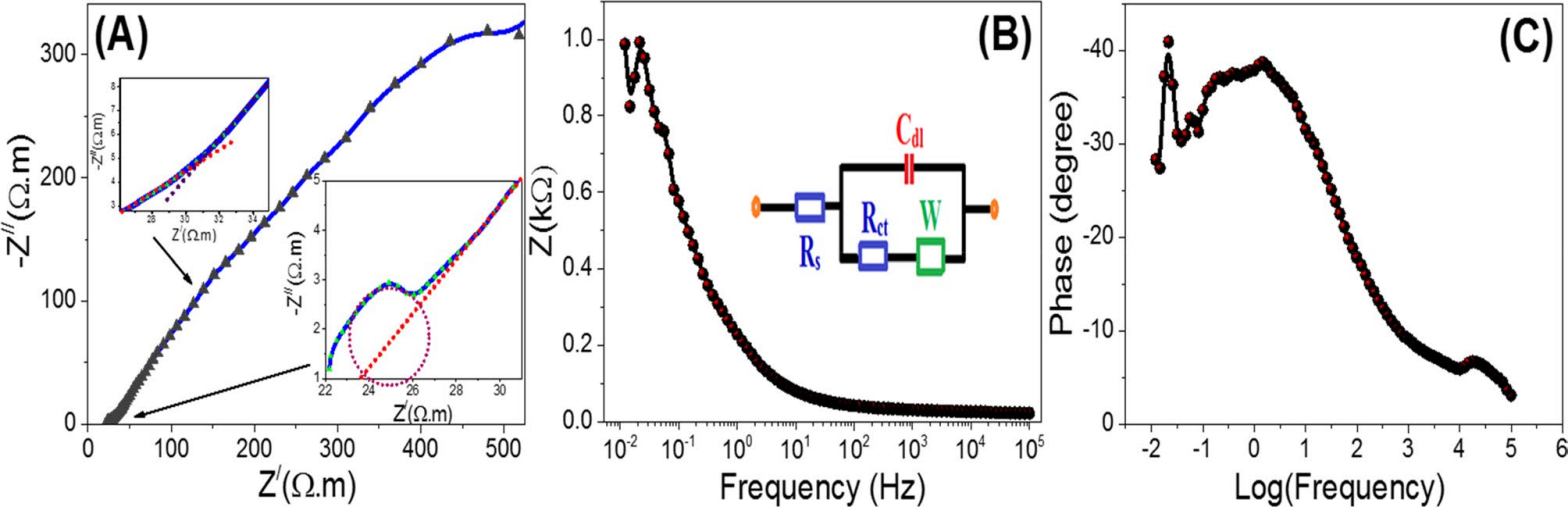
Nyquist plot (**A**), Bode plots (**B**) impedance vs. frequency and (**C**) phase shift vs. logarithm of the frequency for T-LDH/PbI_2_ NC electrode in 0.3 M KOH electrolyte in 0 V (vs. Ag/AgCl) under white light exposure.

Charge carrier dynamics play a vital role in the photocatalytic water-splitting process in deciding the photocatalytic performance of photoelectrode. To investigate the charge carrier dynamics of the T-LDH/PbI_2_ NC electrode, EIS data have been measured by an electrochemical workstation (CHI660E) at room temperature. The photoelectrode was immersed in a 0.3 M KOH electrolyte and the EIS measurements were carried out under illumination at 0 V (vs Ag/AgCl) for a frequency range of 0.01–100,000 Hz. For this photoelectrode, the Nyquist plot is shown in Fig. 12A. This plot exhibited a semicircle at high frequencies due to charge transfer processes in electrode/electrolyte boundaries (charge transfer resistance) and two straight line segments observed at low frequencies with slopes ~ 44° and ~ 69° due to diffusion-controlled processes (Warburg impedance) and additional minimal capacitive activity (double-layer capacitance) as shown in the insets of Fig. 12A. That is to say, mixed diffusion and kinetic controlled routes are illustrated by the EIS data. The results obtained are fitted to a simple equivalent circuit in order to explain the EIS measurements through the hydrogen evolution process. Figure 12B inset displays the suggested Randle equivalent circuit for the simulation of EIS results using the ZSimpWin software (version 3.2; https://echem-software-zsimpwin.software.informer.com/3.2/). This circuit contains the electrolyte resistance (R_s_ = 22 Ω with Fitting error = 0.04965) that can be obtained from Nyquist plot intercept at high frequency, charge transfer resistance (R_ct_ = 4.3 Ω) equals to the semicircle diameter in the Nyquist plot, double-layer capacitance (C_dI_ = 1.472 μF) and Warburg impedance (W = 9.525 × 10^–5^). The reported Rs and R_ct_ values are much smaller than any literature values for LDH-based electrodes, which promoting the PEC hydrogen production^47–49^.

Figure 12B,C presents Bode plots for the T-LDH/PbI_2_ NC electrode, measured at room temperature using 0.3 M KOH electrolyte at 0 V (vs Ag/AgCl). Figure 12B illustrates the total impedance (Z) vs. the frequency, whilst Fig. 12 displays the behavior of the phase vs. the logarithm of the frequency and shows a resistive regime related to the *R*_ct_ at low frequency as well as capacitive contributions related to the *C*_dl_ of the electrode at high frequencies^50^. From Fig. 12C, the maximum phase shift (Ө_max_ in degree), and the frequency at the maximum phase (*f*_*max*_ in Hz), are estimated to be 40.9° in 0.022 Hz. The lifetime of the charge carriers can be estimated from Fig. 12 via the relationship ${\tau }_{n}$ = 1/2π ƒ_max_^50^. The value of the obtained lifetime of the charge carriers for the T-LDH/PbI_2_ NC electrode is estimated to be 7.23 s. The obtained parameters indicate a great reduction in the charge recombination at the electrolyte/electrode interfaces. This also refers to a kinetically facile PEC system, improved ionic conductivity, and electrolytes diffusion through the T-LDH/PbI_2_ NC electrode. Therefore, this photoelectrode showed the highest photocatalytic performance to produce large amounts of H_2_ compared to the previously reported LDH-based electrodes.

##### Effect of applied temperature and calculation of thermodynamic parameters

**Figure 13:**
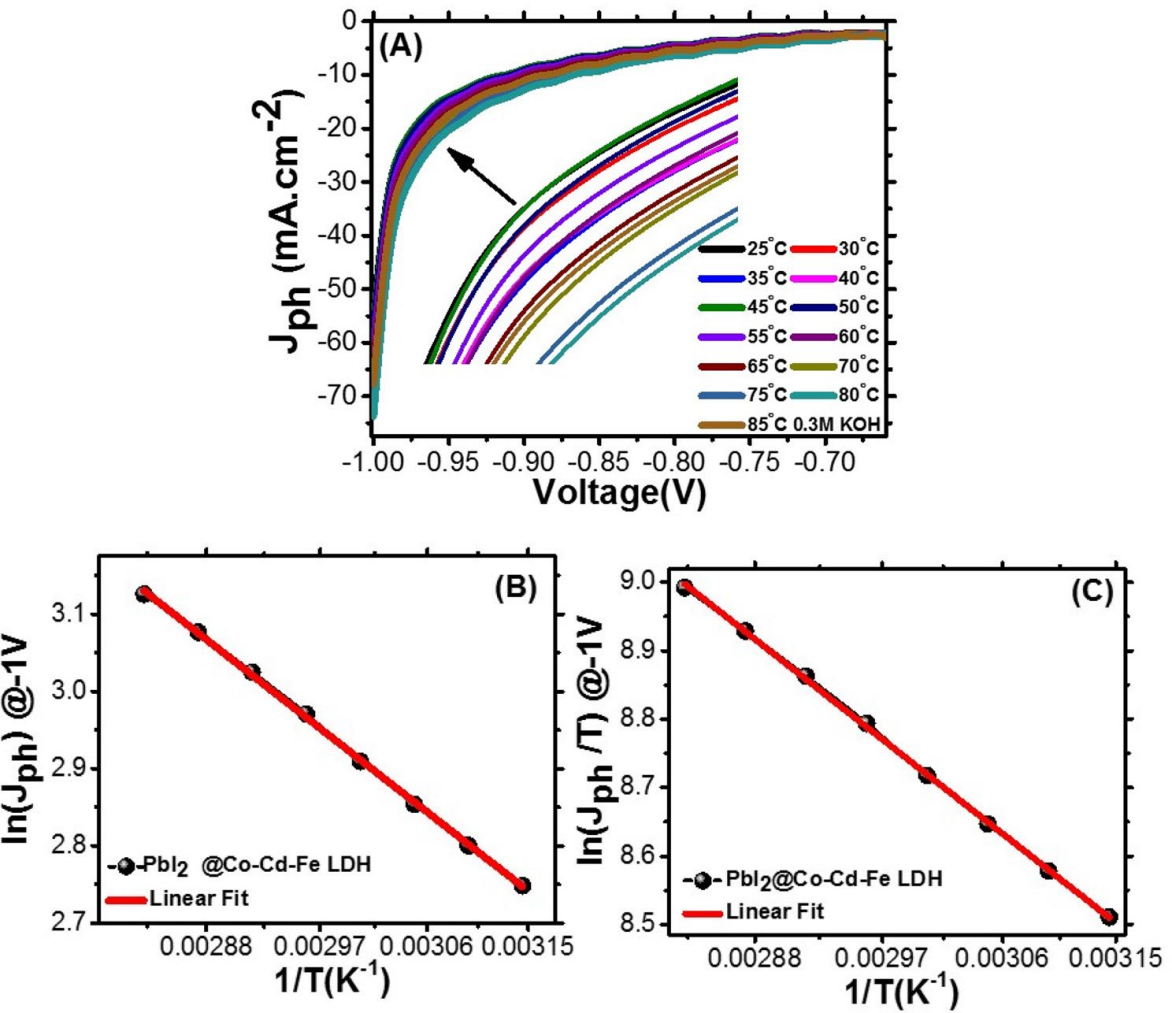
Effect of applied temperature on the PEC J_ph_–voltage curves (**A**), plots of Ln (J_ph_/T) vs. 1/T (**B**), and plots of Ln (J_ph_) vs. 1/T (**C**) for T-LDH/PbI_2_ NC photoelectrode.

The operating temperature is considered a vital parameter that can affect the photoelectrode performance. Figure 13A shows the influence of the applied temperature from 298 to 358 K on the performance of the T-LDH**/**PbI_2_ NC photoelectrode. The J_ph_ increases with increasing the applied temperature to reach its maximum value (73.8 mA cm^−2^) at 253 K. Then, the hydrogen generation rate is increased sharply (1.5 fold) by increasing temperature. i.e., a high reaction temperature will improve dehydrogenation kinetics and release hydrogen at elevated temperature. This increase is due to the decline of the photoelectrode bandgap energy and the increase of the charge transfer rate. According to the Arrhenius plots^50^ (Fig. 13B), the apparent activation energy (Ea) of the HER using T-LDH**/**PbI_2_ NC photoelectrode was calculated to be 9.09 kJ mol^−1^. This value is lower than most of the previously reported values for other LDH-based catalysts^51^. Also, the other thermodynamic parameters such as enthalpy (ΔH*) and entropy (ΔS*) were estimated using the Eyring equation, Fig. 13C. The ΔH* of T-LDH**/**PbI_2_ NC is deliberated from the slope to be 12.99 kJ mol^−1^. While from the intercept, the ΔS* for T-LDH**/**PbI_2_ NC is 78.97 kJ mol^−1^. Table 2 illustrates the PEC performance of our T-LDH**/**PbI_2_ NC photoelectrode comparing with the previously studied LDH-based PEC catalysts^52–58^. As shown in this table, the presented PEC parameters are much higher than the reported performances for the LDH-based catalysts in literatures. As an example, the IPCE% of Ni–Fe LDH/ZnO nanostructures was 82% at 380 nm^52^. Also, ABPE% was 1.24% at 0.62 V for BiVO_4_/CdS/NiCo-LDH^56^. Moreover, J_ph_ of 2.72 mA cm^−2^**/**1.23 V was reported for BiVO_4_/CdS/NiCo-LDH^57^.

**Table 2:** Comparison of the PEC performance parameters of the present work with the previously reported data for LDH-based PEC catalysts.

Electrodes	Performance	References
PbI_2_/Cd Co–Fe LDH	J_ph_ = 53.3 mA cm^−2^ at − 1 V, the number H_2_ moles = 107.53 mmol h^−1^ cm^−2^ and IPCE = 83.8% at 307 nm, 67.3% at 405 nm ABPE% = 4.24% in − 0.58 V and 5.41% in 0.97 V	In this work
Ni–Fe LDH/ZnO nanostructures	IPCE = 82% at 380 nm, J = 1.7 mA cm^−2^, hydrogen rate = 19 mmol/(h cm^2^)	^52^
Co-intercalated LDH composite	IPCE = 1.31% at 365 nm and J_ph_ = 4.67 mA cm^−2^ at 0.8 V vs. SCE	^53^
CdS/ZnCr-LDH	No of H_2_ moles for CdS/ZnCr–LDH (128 mmol h^−1^ g^−1^)	^54^
Co-Mo LDH ultrathin nanosheet	J = 10 mA cm^−2^ at 1.2 V	^55^
Graphene/CoAl LDH@BiVO_4_	IPCE = 52% at 400 nm, and J_ph_ = 2.13 mA cm^−2^ at 1.23 V	^56^
BiVO_4_/CdS/NiCo-LDH	J_ph_ = 2.72 mA cm^−2^ at 1.23 V and ABPE = 1.24% at 0.62 V	^57^
BiVO_4_/Ni_0.5_Fe_0.5_-LDH	J_ph_ = 1.21 mA cm^−2^ in 1.23 V, IPCE = 37.5%	^58^

**Figure 14:**
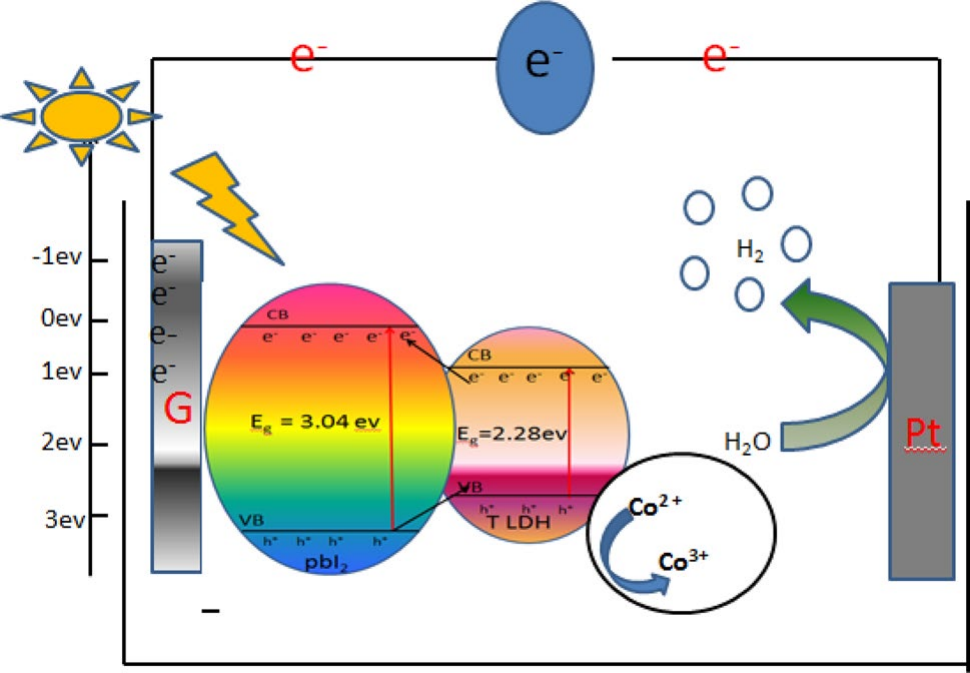
Energy level between T-LDH and PbI_2_ in T-LDH/PbI_2_ NC nanocomposite for PEC water splitting.

Finally, a simple hydrogen generation mechanism is illustrated in Fig. 14 and presented as follows. The incorporation of PbI_2_ nano-semiconductor with a second nano-semiconductor of lower bandgap (Co–Cd–Fe LDH) form a promising photocatalyst to harvest visible light. Some photocatalytic composites can enhance the PEC properties because of the overlapping between the band gaps of two different photocatalysts, which could favor the charge carrier transfer and separation. During applying the external potential bias, photons excite electrons and holes’ separation. The excited electrons migrate from the valence-band (VB) to the conduction-band (CB) of LDH. Then they are transferred to the PbI_2_ catalyst. After that, the transferred electron reacts with the adsorbed H^+^ ion-producing H_2_ molecule. Simultaneously, the residual holes are combined with and removed by the sacrificial reagents of the KOH solution, whereas PbI_2_ avoids the e^–^h^+^ recombination^52^. Finally, PbI_2_ facilities the transfer of the additional CB electrons to Pt and hurries the generation of H_2_ at the active Pt surfaces as shown in Fig. 14.

## Experimental details

### Preparation of photocatalysts

#### Preparation of PbI_2_ electrode

Precipitate PbI_2_ by dissolving both lead (II) nitrate and potassium iodide in distilled water then add a few drops of potassium iodide to the lead (II). As soon as the solutions touch, bright yellow lead iodide is produced. Finally, add the rest of the potassium iodide solution to the lead (II) nitrate. A bright yellow precipitate (PbI_2_) is produced from two clear solutions. PbI_2_ crystals were synthesized by taking the flask containing the PbI_2_ and heat it to near boiling until all of the yellow crystals dissolved and the solution is clear. Then, cool it to room temperature by placing the flask in the lab fridge overnight. After that, Filter is used to isolate the PbI_2_. Carefully remove the tiny crystals on the filter paper to obtain a very beautiful sheet of golden-colored PbI_2_.

#### Preparation of Co–Cd–Fe LDH

NaOH (5 M) was dissolved in 200 mL of distilled water. Another 200 mL aqueous solution of Fe(NO_3_)_3_·9H_2_O (0.1 M), Co (NO_3_)_2_·6H_2_O (0.1 M), and Cd (NO_3_)_2_·4H_2_O (0.1 M) was prepared. This later solution was stirred for 24 h. A pH 10 of the reaction is adjusted by using sodium hydroxide solution. At pH 10, the solution was divided into two solutions; one of them is stirred for 24 h and the second one is put in the autoclave for 3 h. A washing process using DI water is carried out for the resulting precipitate to reduce the pH to 7. Finally, the product is dried at 80 °C for one day.

### Fabrication of T-LDH/PbI_2_ NC

In a general synthesis technique, in-situ growth of the metal cations, typically, NaOH (5 M) in 200 ml of distilled H_2_O is prepared. Another solution of Fe (NO_3_)_3_·9H_2_O (0.1 M), Co (NO_3_)_21_·6H_2_O (0.1 M), Cd (NO_3_)_2_·4H_2_O (0.1 M), and 2.5 g PbI_2_ was prepared. This later solution was stirred for 24 h. A pH 10 of the reaction is adjusted by using the sodium hydroxide solution. After reaching pH 10, the solution was remained under continuous stirring for 24 h. A washing process using DI water is carried out for the resulting precipitate to reduce the pH to 7. After washing, a drying process is carried out at 80 °C for one day.

### Fabrication of the PEC photoelectrodes

Three different photoelectrodes are fabricated to be used for the PEC hydrogen production. 3% of each photocatalyst (PbI_2_, Co–Cd–Fe LDH, and T-LDH/PbI_2_ NC) is mixed with 3% of C_6_H_4_ (CO_2_C_4_H_9_)_2_ plasticizer (DBP, 99.8%) and 3% of (C_2_H_3_Cl)_n_ (PVC, 99.8%) in the least amount of (CH_2_)_4_O (THF, 99.9%). DBP, PVC, and THF were obtained from the Egyptian Middle East company. The three products of the mixing process are moved to three 5 cm-Petri dishes. The mass of each batch is 0.35 g. Then, the three Petri dishes are left to dry and sealed off with three filter papers. By fixing the amount of THF and carrying out the drying process for one day, the thickness of each photoelectrode is fixed to be 200 μm.

### Characterization of different photocatalysts

The XRD patterns of PbI_2,_ Co–Cd–Fe LDH, and T-LDH/PbI_2_ NC were obtained by Philips X^’^Pert_1_-MRD X-ray diffraction (λ_CuKa_ = 0.15418 nm). Samples morphology is investigated using a field-emission scanning electron microscope (FESEM,HRTEM, Zeiss SUPRA/55VP with GEMINI/column). (Fourier Transform Infrared Spectroscopy (FTIR) was performed by A Shimadzu-FTIR-340-Jasco spectrometer to obtain the important functional groups of the samples. Finally, the optical absorbance behaviors of the products are investigated by Lambda 900-UV/Vis/IR Perkin Elmer spectrophotometer up to 1200 nm.

## Conclusion

In summary, a novel technique for loading Cd–Co–Fe-LDH/PbI_2_ has been introduced to fabricate an efficient nanocomposite photocatalyst. For comparison, the different properties of PbI_2_, Co–Cd–Fe LDH, and T-LDH/PbI_2_ NC were investigated using various instruments; XRD, FTIR, HR-TEM, FE-SEM, and UV–Vis–IR spectrophotometer. The growth of LDH on PbI_2_ prevents the agglomeration of LDH nanoparticles and allows the distribution of the particles to increase the surface area and decrease the particle size. Loading of LDH narrows the bandgap of PbI_2_ from 3.04 to 2.53 eV for T-LDH/PbI_2_ NC, which prolongs the lifetime of the photo-induced electrons. Consequently, the application of T-LDH/PbI_2_ NC improves the PEC H_2_ production rate to reach 107.53 mmol h^−1^ cm^−2^ and IPCE% to reach 83.8% in 307 nm and 67.3% in 508 nm. The ABPE% reach its maximum value (4.24%) at − 0.58 V and (5.41%) at − 0.97 V. To the best of our knowledge, the performance of T-LDH@PbI_2_ NC as a PEC catalyst is higher than any previously reported LDH-based photocatalysts. The effects of the operating temperature and monochromatic illumination on the PEC performance were studied. Also, the electrochemical surface area, thermodynamic parameters, and Tafel slopes are calculated to label the hydrogen evolution mechanism. The T-LDH/PbI_2_ NC photoelectrode displayed lower Tafel slopes and a much higher electrochemical surface area compared to T-LDH and PbI_2_ electrodes. Moreover, the activation energy of T-LDH/PbI_2_ NC was 9.09 kJ mol^−1^, which was lower than any previously reported value for LDH catalysts. This study has provided a new viewpoint to design highly active photocatalysts for solar light-driven H_2_ production.
